# Failed medial buttress plating of a femoral neck fracture salvaged with a Pauwels osteotomy: Case report and review of literature

**DOI:** 10.1016/j.tcr.2021.100569

**Published:** 2021-12-08

**Authors:** Niels Hendrik Bech, Peter Kloen

**Affiliations:** Department of Orthopedic Surgery, Amsterdam University Medical Center, the Netherlands

**Keywords:** Femoral neck fractures, Fracture healing, Osteotomy

## Abstract

Two young patients with a Pauwels type 3 femoral neck fracture were treated with cannulated screws and the addition of an anteromedial buttress plate on the femoral neck. Both developed a non-union necessitating a salvage procedure. A Pauwels' osteotomy led to uneventful and complete healing in both patients.

The purpose of this report is to describe the current literature on anteromedial buttress plating in femoral neck fractures and discusses a reproducible hip preserving salvage option when a non-union develops.

## Introduction

A femoral neck fracture in the young patient (<65) is often the result of high energy trauma [1]. Pauwels has classified this fracture in three types according to the angle of the fracture [2]. A Pauwels type 3 fracture has a vertical angle greater than 50 degrees. Because of the high vertical shear forces during loading the Pauwels type 3 fracture is more likely to result in fracture displacement, varus collapse and non-union [3].

Historically, for fixation of these femoral neck fractures several osteosynthesis options are possible. Most often cannulated screws are used. For the parallel configuration of the screws; most people advocate an inverted triangle with as much spread as possible between the screws, with no screw starting below the level of the lesser trochanter [4]. The use of washers increases biomechanical holding power. Others have used two parallel screws with a third more horizontal screw that theoretically resists shearing forces. Surgeons can choose between partially threaded or fully threaded screws. Less often, a dynamic hip screw with de-rotational screw or locking type plates are used. These methods work well for Pauwels 1 and 2 fractures. However, a Pauwels 3 fracture remains a challenge.

Mir and Collinge added a medial buttress plate to the femoral neck [5]. This functions as an anti-glide plate neutralizing the shear forces of the Pauwels type 3 fracture. Small series have shown good results [6], [7], [8]. Not all series used the same technique with variations being plate-type (locking, one-third tubular or anatomic) and plate position (medial/anteromedial).

Our early experience was complicated by two failures leading to a non-union. The purpose of this paper is to describe the literature on medial buttress plating of the femoral neck fracture and to present two salvages of our early failures.

## Case 1

**Fig. 1:**
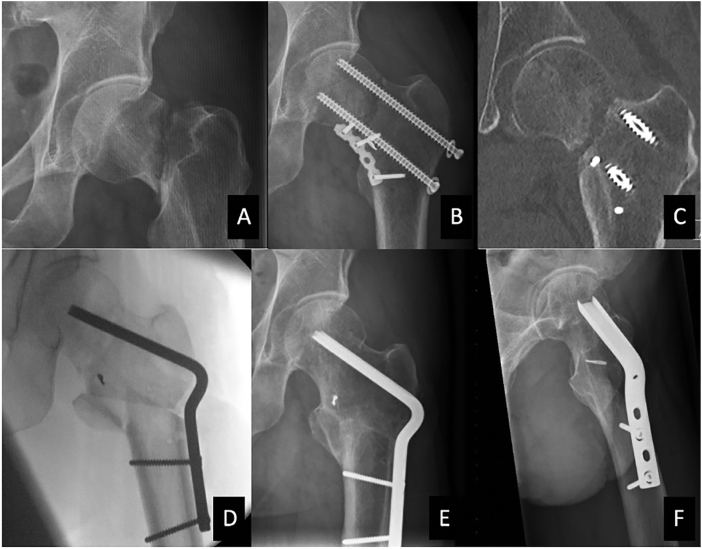
A: AO type 31B2.3 fracture of the left hip; B + C: X-ray and CT scan 2 months after surgery without signs of fracture healing; D: Perioperative imaging of the Pauwels osteotomy; E + F: Complete healing and union, 17 months after Pauwels osteotomy.

A 54-year-old male sustained an AO 31B2.3 fracture of the left hip (Pauwels type 3 fracture; Fig. 1). Mechanism of injury was a fall with a moped. Within 6 h we operated the patient in the supine position under general anesthesia. After a Smith-Peterson approach and capsulotomy, a pointed reduction clamp kept the fracture reduced and compressed. Blood supply to the femoral head was confirmed by drilling a small hole in the head that showed bleeding. The fracture was fixated with three fully threaded cannulated screws (7.3 mm) with washers in an inverted triangle configuration. Next, we inserted a 6-hole 3.5 mm titanium reconstruction LCP plate (DePuy Synthes, Amersfoort, the Netherlands).

The plate was contoured and positioned on the anteromedial side of the femoral neck; between the 5 and 6 o'clock position. When testing the hip prior to closure an audible clicking was noted on flexion and rotation of the hip. We thought the posterior cannulated screw was malpositioned with its middle section posteriorly outside of the neck. However, after removing this screw, the click persisted. We noticed that the proximal end of the anteromedial buttress plate was impinging on the acetabulum. The plate was removed, and replaced by a shorter plate (4-hole), more distal and medial (Fig. 1). The posterior cannulated screw was not replaced because we were not sure if it had been malpositioned. Replacing it in a more anterior position was tried but the guidewire kept slipping into the prior entry point. We felt the fixation with 2 screws and a plate was strong enough. He started toe-touch weightbearing for 6 weeks. At 6 weeks, he started partial weightbearing but reported pain after 2 months. Discussing the options of waiting versus early intervention (Pauwels' osteotomy) versus a total hip arthroplasty, the patient elected a Pauwels' osteotomy. We were comfortable offering a Pauwels' osteotomy given our large experience with this technique. This second surgery was 60 days after injury. A lateral closing wedge of 35 degrees was removed. A 5-hole 120-degree angular blade plate (85 mm) was inserted. At 6 weeks the radiographs showed early consolidation of both the femoral neck non-union and the Pauwels' osteotomy. He started partial weight bearing at 6 weeks, and full weight bearing at 3 months. At 4.5 months he was pain-free with full range of motion of his hip and a normal gait. He did not return for follow up until 29 months. Radiographs showed a healed osteotomy and a healed nonunion (Fig. 1). The patient reported 1 on the NRS pain scale (10 being worst pain possible) and 95 points (maximum 100) on the Harris Hip score (HHS).

## Case 2

**Fig. 2:**
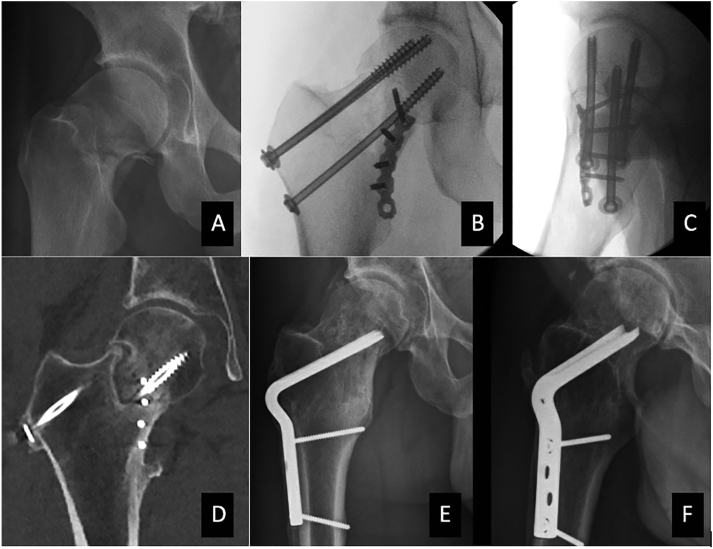
A: AO type 31B2.1 fracture of the right hip; B + C: Perioperative imaging of the anteromedial plate; D: CT scan that shows development of non-union 7 months after surgery; E + F: Complete healing 22 months after Pauwels osteotomy.

A 43-year-old male sustained an AO 31B2.3 hip fracture (Pauwels type 3 fracture; Fig. 2) in a bicycle accident. Within 6 h, we operated the patient in the supine position under general anesthesia. After anatomic reduction via a Smith-Peterson approach, we placed a 5-hole LCP reconstruction plate on the anteromedial side of the femoral neck. Blood supply to the femoral head was confirmed by drilling a small hole in the head that showed bleeding. Three partially threaded cannulated screws (7.3 mm) with washers were inserted. He was toe-touch weightbearing for 6 weeks. Radiographs at 6 weeks suggested bony bridging. He advanced to partial weightbearing at 6 weeks. At 7 months, a CT-scan showed a nonunion (Fig. 2). An MRI at that same time did not show evidence of avascular necrosis. A Pauwels' osteotomy was offered. A lateral closing wedge of 20 degrees was removed. A 110-degree angular blade plate (85 mm) was inserted. At 6 weeks post-operatively, he was partial weight bearing. At 3 months follow up he was fully weight bearing. At 21 months he returned for a follow-up visit. He was pain-free with full range of motion with excellent outcome scores (NRS 1, HHS 94). Radiographs showed consolidation (Fig. 2), Brooker grade 1 heterotopic ossification without avascular necrosis or arthrosis.

## Discussion

Medial buttress plating to augment fixation in a femoral neck Pauwels 3 fracture is a new technique [5], [6], [7], [9]. Mir and Collinge introduced it in a hypothetical paper explaining the concept of an anti-glide plate converting high shearing force into compression along a reduced anteroinferior fracture spike [5]. Since their description, two case series and a case report have been published [6], [7], [8].

The two series published represent 56 patients [6], [7]. Zhuang et al. placed the buttress plate anteromedial and reported non-union rate of 0%; Ye et al. positioned the plate medial and reported non-union rate of 11%. In Ye et al.'s study, an additional 3 patients (11%) had screw back out and/or plate breakage suggesting delayed healing or maybe even a non-union [6]. This would increase their failure rate to 22%.

In the case report of Marchand et al. the fracture healed but the patient developed arthrosis due to impingement of the plate [8].

Promising and out-of-the-box techniques such as buttress plating for femoral neck fixation have emerged. However, in our hands this method has not proven a fail-safe approach. We believe it is still important to attempt promising advanced techniques, but be prepared to implement a classic salvage treatment as shown herein (Pauwels' osteotomy).

## Declaration of competing interest

The authors declare that they have no conflict of interest.
